# Functional testing in animal models of spinal cord injury: not as straight forward as one would think

**DOI:** 10.3389/fnint.2013.00085

**Published:** 2013-11-26

**Authors:** Karim Fouad, Caitlin Hurd, David S. K. Magnuson

**Affiliations:** ^1^Faculty of Rehabilitation Medicine, Centre for Neuroscience, University of AlbertaEdmonton, AB, Canada; ^2^Friends for Michael Chair in Spinal Cord Injury Research, Kentucky Spinal Cord Injury Research Center, University of LouisvilleLouisville, KY, USA

**Keywords:** recovery, spinal cord injury, compensation, locomotion, grasping

## Abstract

When exploring potential treatments for spinal cord injury (SCI), functional recovery is deemed the most relevant outcome measure when it comes to translational considerations. Yet, assessing such recovery and potential treatment effects is challenging and the pitfalls are frequently underestimated. The consequences are that in many cases positive results cannot be reliably replicated, and likely treatments that appear to lack effects have been dismissed prematurely. In this article we review the relationships between lesion location/severity and functional outcomes with specific consideration given to floor and ceiling effects. The roles of compensatory strategies, the challenges of distinguishing them from *bona fide* recovery, and of comparing function to pre-injury levels given the variability inherent in animal testing are discussed. Ultimately, we offer a series of considerations to enhance the power of functional analysis in animal models of SCI.

Over the last several decades a plethora of studies have been conducted in animal models of spinal cord injury (SCI). A simple PubMed search identifies over 10,000 publications in rats and mice. Many of these studies were targeted to understand and to overcome the mechanisms that inhibit neurite (out) growth in the adult mammalian central nervous system (CNS). Others explored possible strategies to reduce secondary damage following spinal insult or investigated the adaptive changes occurring in response to SCI. Ultimately, all this research has one common goal: to discover ways of promoting functional recovery following SCI. Despite the increasingly apparent realization that direct translation of functional recovery from feline and rodent models to human is difficult (Rosenzweig et al., 2009; Kwon et al., 2010, 2013), recovery remains the strongest incentive to translate a treatment from any animal model to clinical trials. In this review we define “recovery” as the combination of functional restoration and functional compensation or the use of alternative approaches to perform a task.

Within the vastly expanding field of SCI research, a variety of animal models have been utilized. These models have involved different species (focusing on rodents, felines and primates) and have employed different lesion methods (including contusion, compression and laceration) at different locations of the spinal cord with varying severities. As a logical consequence, these different approaches resulted in very different functional outcomes that necessitated the development of a variety of behavioral tests. Many of these tests were similar to each other (e.g., Montoya staircase test and single pellet reaching, horizontal ladder test and grid walk), others were very different from each other, although used for similar lesion models (e.g., incline plane, foot placing, kinematics). The interpretation of this array of tests has been further complicated by laboratory-dependent modifications, resulting in data sets that are difficult to compare between laboratories and treatments. An important breakthrough was achieved when the Basso, Beattie, Bresnahan (BBB) Open Field Locomotor Scale was introduced (Basso et al., 1995). The BBB is a scale that was designed to evaluate open field locomotion following moderate contusion injuries in rats. Personnel from many laboratories have been trained to utilize this outcome measure and the BBB now provides a “universal language” of hindlimb recovery in rat models and, more recently in mouse models using the Basso Mouse Scale (Basso et al., 2006). Reporting hindlimb function using the BBB Scale has become an “unwritten” requirement for any publication or grant application that uses a rat model of SCI. The enthusiasm for, and widespread use of, the BBB Scale has unfortunately also resulted in frequent misuse and/or miss-interpretation of results. The BBB Scale was developed based on a standard T9 contusion injury in adult rats, however its popularity has lead to its being used for a huge variety of lesion models and therapeutic approaches, including excitotoxic and ischemic lesions (Magnuson et al., 1999; Takeda et al., 2011). The inappropriateness of the BBB for a variety of lesion models is not the only issue with the scale; it has also been shown that the scale is not linear (Schucht et al., 2002). In other words, animals are not distributed evenly along the scale when lesion severity is applied randomly. Instead, there are a few points on the scale where rats have been shown to “cluster”, namely at ratings of 8 and 14. This is an issue when comparing the effectiveness of potential treatments because this clustering reduces the resolution and sensitivity of the scale to assess recovery. In addition, the functional differences between points on the scale are very minor for some and are critical for others. For example, the change from 3 to 5 involves “extensive movement of two joints” to “slight movement of two joints and extensive movement of the third”, while a change from 8 to 10 involves “sweeping with no weight support or plantar placement of the paw with no weight support” to “occasional weight-supported plantar steps; no FL-HL coordination”. In summary, despite the tremendous value that the BBB Scale has provided to the field, there is no standard procedure to judge and quantify recovery in the wide variety of animal models and therapeutic approaches currently being studied. The variety of models and outcome measures are a constant source of discussion, disagreement and misinterpretation. Thus, an answer to the common question (asked not only in the field of SCI), of why many treatments that work in animal models do not in the clinic, may be the “inappropriateness” of our animal models. But the answer is not that simple, and there is more to the story. Considering that variability is an even greater challenge in the clinical setting than in animal models, it is often suggested that it contributes to the modest translational success in treatments for SCI. Significant results in well-controlled animal models might not be strong enough to show statistical effects in a clinic trial. When a successful treatment is identified, the variability in the patient population could be used to advantage by helping to delineate which injured population, based on age, gender, injury severity and location, is most likely to benefit from a treatment. This would be important information because treatment-induced recovery in a rodent model does not necessarily mean that primates and individuals with SCI will recover to a similar degree (or to any degree) given the same therapy.

Behavioral testing in animal models can produce false positive, non-repeatable or false negative findings. In this review we will address some of the challenges of functional testing in rats with cervical or thoracic spinal cord injuries, a difficult task even without testing the effects of potential therapeutic treatments.

## Lesion size and recovery

**Figure 1:**
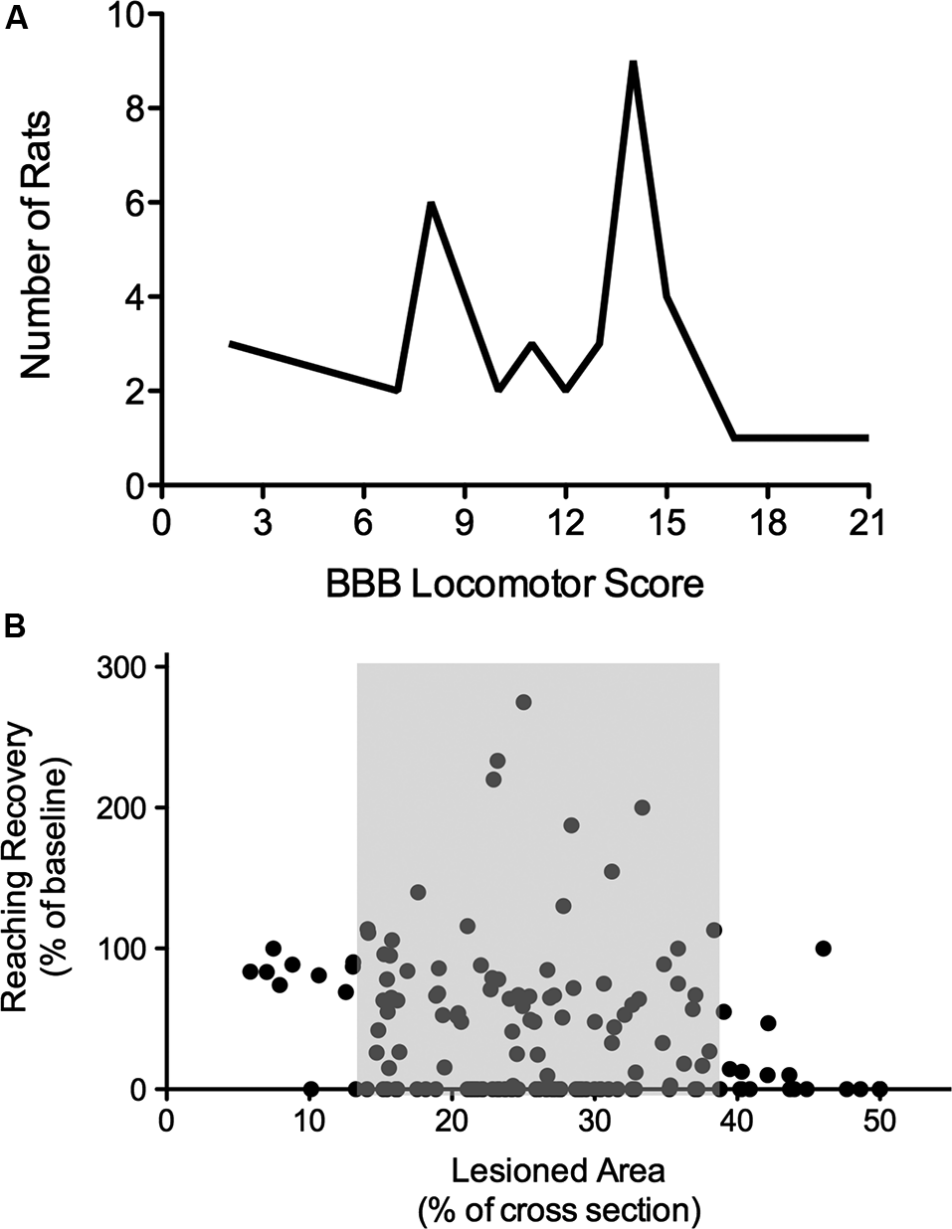
**Nonlinearity of lesion severity and functional recovery. (A)** Clustering of animals in the BBB locomotor score. Although T8 lesion severity and location (dorsal versus ventral) were applied randomly, two scoring points were assigned most frequently, indicating thresholds in the BBB scale. Adapted from Schucht et al. (2002). **(B)** Correlations of lesion and reaching success. Total lesioned area at C4 was not correlated with final single pellet reaching success. Success is consistently high with small lesions and low with extensive lesions, however, when approximately 14–38% of the dorsolateral spinal cross section is lesioned, there is substantial variability in reaching recovery (grey area). Adapted from Hurd et al. (2013).

It is not necessarily surprising, but frequently ignored, that spinal lesions with increasing severity do not always result in comparable decreases in function. In other words, the percentage of spared spinal tissue, or even spared white matter, is not linearly related to an inverse decline in functional recovery. Such non-linear relations between injury and recovery can be found following thoracic injuries and locomotor function (Schucht et al., 2002) as well as following cervical lesions and paw function (Hurd et al., 2013; Figure 1). A typical picture that arises is that lesions sparing greater than 30–40% of tissue at the epicenter (area of white matter in a cross-section) affect motor function very little, but with increasing lesion severity a substantial drop in function occurs along with a considerable increase in variability. This has a huge impact on the evaluation of function and of treatments. For example, recovery might not be detectable regardless of the mechanism of action of a potential treatment, whether by restoring conduction across the lesion site, promoting plasticity, or by facilitating tissue sparing. Tests might not be sensitive enough throughout the entire range of lesion severity.

There are many reasons for a non-linear relationship between lesion size and recovery. Tracts are not evenly distributed within the spinal cord, there appears to be a threshold proportion of a particular tract that can be lost before detectable deficits occur or before function is not able to be recovered (Figure 1B; Loy et al., 2002; Hurd et al., 2013). Most importantly, motor function is not controlled by a single, or even a few tracts, but by an adaptive network of descending tracts with overlapping function and by spinal networks including reflex pathways, short and long propriospinal pathways and pattern generating (rhythmic) circuitry. This constellation allows for a high degree of neural circuitry-based compensation after injury of the nervous system, contributing greatly to the non-linearity of recovery. Moreover, compensation is also observed on a behavioral level and might well be linked to activity-driven plasticity in the entire CNS.

## Compensation and motor recovery

Functional recovery following various injuries to the nervous system includes compensatory behavior. Animals may recover function in motor behaviors such as walking, swimming, reaching and ladder walking, but they tend to accomplish the task or behavior using different strategies than they used before an injury. In other words, animals may compensate and/or relearn the task or behavior. Since behaviors such as single pellet grasping and locomotion are characterized by stereotypic movement patterns, post-injury adaptations in these patterns can be assessed and have been shown to contribute to recovery (e.g., Ballermann et al., 2006; Gharbawie et al., 2006; Smith et al., 2006a). However, “true” restoration/recovery following SCI would require restoring the pre injury motor pattern. Thus, compensation would allow the detection of recovery in quantitative outcome measures (e.g., how fast can a rat walk or how many pellets can it grasp over a period of time) but not necessarily in sensitive qualitative outcome measures (e.g., scores).

Compensation can take many forms, ranging from slight modifications to a complete replacement of a motor process. For example, uninjured rats swim bipedally, relying completely on their hindlimbs for propulsion and using their forelimbs only for steering and obstacle avoidance. Acutely after a SCI, rats again swim bipedally, however they compensate for hindlimb plegia by relying completely on their forelimbs for forward motion (Smith et al., 2006a). Thus, if deficits are significant, compensatory strategies must be acquired to succeed in a given task. While many of these compensations are obvious, as in the example of swimming, many are subtler and may not be accurately characterized by the functional assessments being used. In such cases additional outcome measures such as electrophysiological and/or kinematic approaches are needed to assess compensatory mechanisms (Ballermann et al., 2006). Using swimming as the example again, if motor recovery is assessed using only the number of pool laps completed, compensation with their forelimbs will allow even severely injured rats to complete 20–30% of “normal” without the use of their hindlimbs at all (Smith et al., 2006b; and unpublished observations).

A well-established example where a modification of a motor function following SCI can be observed is grasping. It has been described for many years that cortical input is needed for precision grip in primates (Muir and Lemon, 1983). Similarly, in rodents with cervical SCIs that ablate the Corticospinal and/or Rubrospinal tract fibers, reaching and grasping is compromised in that precise grasping of small objects is hindered (Kanagal and Muir, 2007; Stackhouse et al., 2008; Morris et al., 2011). In the case of skilled reaching, compensation may occur at any point in the reaching sequence. When rats are tested following CNS injury, for example by presenting small sucrose pellets, they can perform this task quite well by adopting a compensatory raking or scooping strategy when unable to flex and close their digits (Gharbawie et al., 2006). This strategy is used throughout a large spectrum of lesion severities and can contribute to the nonlinear relation between lesion size and success rate in pellet retrieval (Hurd et al., 2013). For example, if reaching success is quantified as the number of pellets retrieved as a percent of the total number of pellets presented, a rat that adopts a new reaching strategy such as scooping may appear more successful than a rat that maintains their pre-injury reaching strategy following a lesion. Interestingly, rats from the same breeding colony have been shown to adopt different compensatory reaching strategies, naturally contributing to variability in reaching performance (Gholamrezaei and Whishaw, 2009). The use of such gestures or compensatory strategies suggests that these movements should be scored in order to be able to illustrate and quantify possible lesion or treatment effects. The advantages and limitations of scores and qualitative analyses in general will be discussed in the next section.

Drastic examples where a function is completely replaced can be seen during walking or swimming following complete SCI. For example, when locomotion is defined as a task to get from point A to B, rats show a great ability to recover by compensating. Assuming that the floor surface offers sufficient traction, rats can reach surprising speeds by just using their forelimbs. As mentioned earlier, during swimming uninjured rats rely on their hindlimbs only, while post-injury propulsion is taken over by the forelimbs. In the clinic we see a similar picture, with assistive devices being part of compensatory strategies. Wheelchairs can completely compensate for the loss of leg function and can even allow a wheelchair athlete to finish a marathon significantly faster than any running athlete. Thus, compensation allows for tasks to be accomplished, but this also introduces a major challenge. Studies into functional recovery following stroke and more recently also following SCI have shown that in order to maximize recovery, extensive use of affected neural circuitry is essential (Nudo et al., 1996; Bareyre et al., 2004). It also appears that this needs to be accomplished relatively early after injury due to a perceived period of increased neuroplasticity, a “window of opportunity”, present following injuries to the CNS, allowing a level of “rewiring” and optimization of spared circuitry (Taub et al., 2006; Krajacic et al., 2009). In the field of stroke research, constraint-induced movement therapy, where compensation using the less-affected arm/paw is restricted by restraint, is a commonly used and successful rehabilitative strategy that has been translated from animal models to the clinic (DeBow et al., 2003; Taub, 2012; Zhao et al., 2013). In conclusion, if compensatory mechanisms are quickly introduced or allowed following CNS injury, “true” restoration of lost function may be limited. Activation of spared circuitry is essential to recover as much “original” function (i.e., restoration) as possible.

## Scoring recovery

 The prominent role of compensation in the recovery of function raises important issues when considering how recovery should be evaluated. Two main approaches could be chosen: qualitative or quantitative testing. As discussed earlier, qualitative scores of movement patterns are popular approaches that have been used for evaluating locomotion (Wrathall et al., 1985; Basso et al., 1995) and grasping (Whishaw and Pellis, 1990) in rats with various injuries to the CNS. These scoring systems normally compare a movement pattern to that of normal animals. An obvious challenge with this approach is that one cannot necessarily determine whether compensation occurred and if the “abnormal” execution of a movement pattern is actually beneficial to task performance. For example, rats with an abnormal reaching score might still achieve success rates of pellet retrieval similar to pre-injury.

Evaluating recovery quantitatively (e.g., speed of walking, reaching success) can also be deceiving, as rats might walk well and fast with forelimbs only, walk better on an elevated narrow beam without hind leg movements (uncoordinated movements tend to push the animal off the beam), or reach better by using a compensatory grasping strategy such as scooping. In conclusion, it is necessary to evaluate recovery both qualitatively and quantitatively, evaluating both changes in how the task or behavior is executed and the ultimate success in the task, in order to accurately and sensitively determine the influence of a treatment on recovery.

## Hands on challenges of animal testing

Accurate and sensitive quantification of functional recovery following SCI is difficult due to the relatively high variability observed even when biomechanically similar injuries are generated. In this section we explore some of the factors that may be responsible for that variability.

### Lesion variability

Although it is thought that lesions can be reliably reproduced in animal models, significant variability can be found throughout lesion models/approaches. Even the use of devices engineered to deliver impacts that are mechanically identical can result in a wide range of lesion severities. Differences in the arrangement of the vasculature relative to the impact site and very minor changes in positioning of the device can contribute to variability. The hormone status of the animals, their level of hydration and even the blood sugar level could influence aspects of the acute injury (Kwo et al., 1989; Gruner, 1992). Anesthesia is also critically important, both in terms of inducing variable levels of neuroprotection, for example if Ketamine is used, as well as by adding variability in neuronal responses to the physical component of the impact. Furthermore, because secondary damage is a process involving inflammation, the immune status of the individual animals could contribute to variability in lesion size.

### Animals

It is now well documented that different rodent strains show different specific behavioral trademarks, including wide differences in motor control (Webb et al., 2003). Reaching for food pellets is a great example of strain-dependent differences in motor skills. Hooded long Evans are superior to all other strains on the single pellet skilled reaching task, and Lewis rats appear to perform even worse than the most inbred of experimental rat strains, the Fischer (Nikkhah et al., 1998; VandenBerg et al., 2002). An additional concern is that differences in the rate of learning a motor task and in levels of final success can be found even within a single strain of rats. For example, when rats of the same strain are purchased from a different supplier but trained by the same experimenter at the same time (O’Bryant et al., 2011).

### Variables influencing behavior

**Figure 2:**
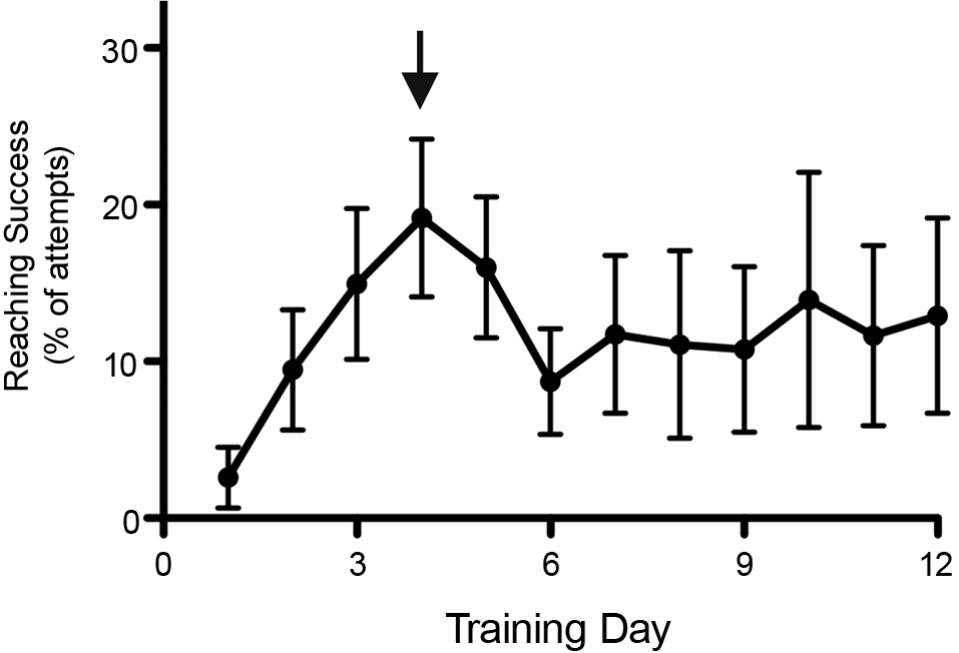
**Decreased performance in behavioral tests by rats in the vicinity of stress.** Reaching performance dropped substantially when a second (independent) group of rats housed in the same room underwent surgery (as indicated by the arrow), an intus cella effect. Reaching performance remained low for over a week. Data is shown as mean ± SEM, *n* = 12.

Animal behavior is influenced by many variables that have to be controlled and/or assessed whenever possible. For example, animals are very stress sensitive and precautions should be taken to avoid stress-inducing factors such as changing the experimenter, irregular activities including testing at different times of the day as well as excessive noise during testing. A case in point: In one of our studies, rats were tested daily in a single pellet reaching task and when a second (independent) group of rats housed in the same room underwent surgeries, the resulting stress caused a substantial drop in reaching performance that lasted over a week (Figure 2). This troubling observation suggests that the animals undergoing surgery expose the non-surgical animals to stress-inducing sounds (Kurejova et al., 2010) or smells (Valenta and Rigby, 1968; Stevens and Köster, 1972; Mackay-Sim and Laing, 1980) that can influence their reaching behavior.

**Figure 3:**
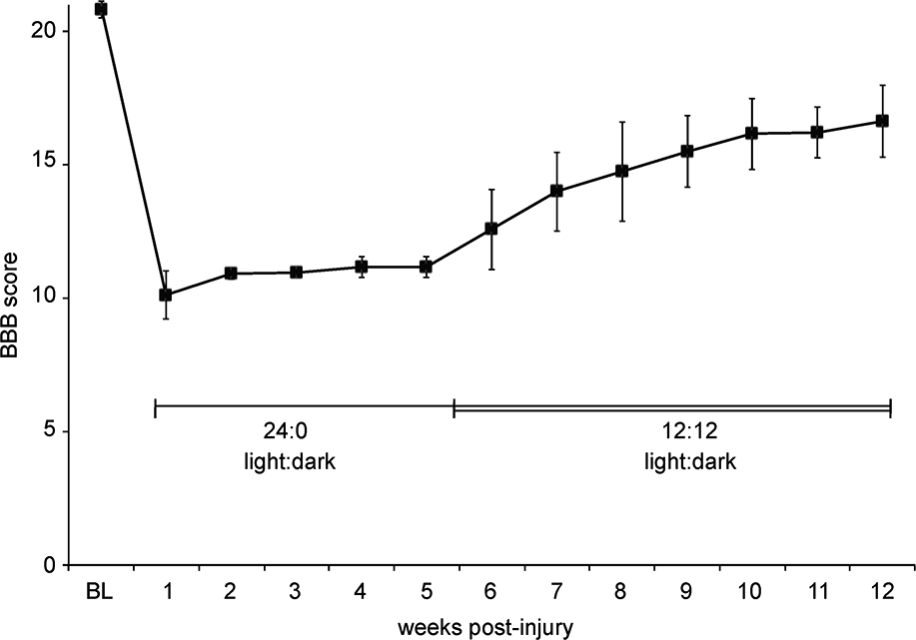
**Abnormal light: dark cycle impairs locomotor recovery.** Graph shows BBB scores over time for a group of eight female SD rats housed in pairs. Due to a malfunction, we believe that the animals were in constant light (24:0, light:dark) starting 1 day post-injury until the 12:12, light:dark cycle was restored at 5 weeks post-injury. Data is shown as mean ± SD.

Circadian rhythms and the day-night cycle can also strongly influence stress levels (Dauchy et al., 2010), and therefore performance of rats in behavioral testing, to the point that many groups invert the day/night cycle of their colony to examine rodents during the dark cycle when they are most active and alert. A case in point: Several years ago we experienced an unexpected example of how critical housing conditions are to behavioral recovery. At this time our animal housing facility was using electromechanical devices to control the lighting in the room where post-injury animals were maintained. This device malfunctioned and we believe the animals were housed in constant light for the first five weeks of the study (24:0 light:dark cycle; we don’t know when the malfunction occurred). When this was noticed by a graduate student the device was replaced and the animals were immediately switched to a 12:12 light:dark cycle. The BBB scores of these young female SD rats rose dramatically over the next several weeks, despite having already plateaued, albeit at lower than anticipated scores (Figure 3). These animals were 175 g at the time of injury and received 12.5 g-cm NYU injuries at T10 under Nembutal anesthesia. We hypothesized that this jump in hindlimb function was due to decreased stress and increased in-cage activity when the light:dark cycle was returned to normal.

Less influential than the circadian rhythms but still a contributor to variability in experimental outcome is the time of the year when an experiment is performed. For example, O’Bryant et al. (2011) found that animals shipped in the summer and trained in the fall had lower rates of reaching success compared to rats trained in other seasons.

### Spontaneous activity

Activity in the home cage is an essential contributor to recovery following SCI. In the case of locomotion it has been proposed that walking in the home cage promotes recovery and may maximize recovery (ceiling effect), the likely cause for the frequently underwhelming results of treadmill training in rats (Fouad et al., 2000; Heng and de Leon, 2009; Kuerzi et al., 2010). Similarly, in-cage forelimb use can influence reaching performance and/or the effect of reaching training Under normal circumstances single pellet reaching training improves pellet reaching performance following cervical spinal cord injuries (Girgis et al., 2007; Krajacic et al., 2009). However, we found that supplying sunflower seeds to the animals in their home cages, no training induced recovery could be observed (unpublished observations).

**Figure 4:**
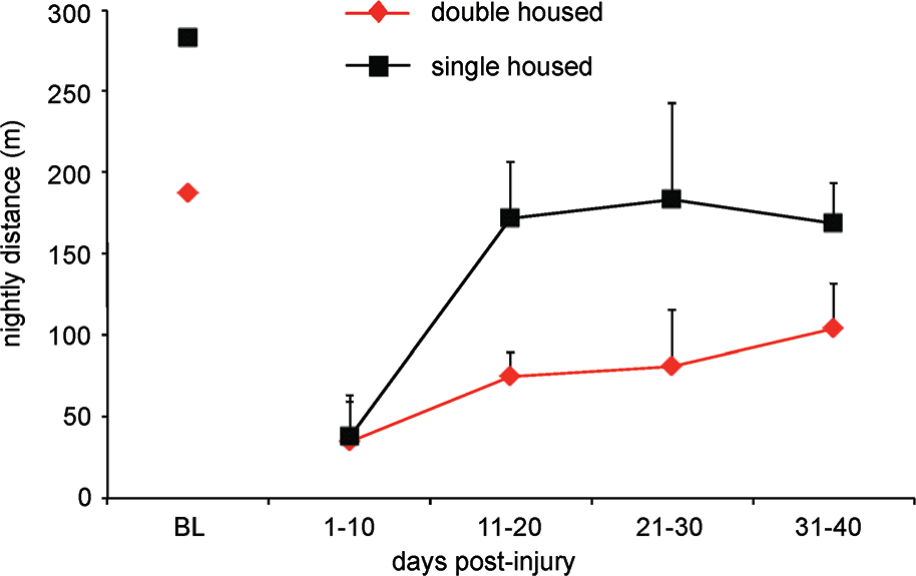
**Rats traverse a significantly greater distance when housed in pairs as compared to single housed.** Graph shows total distance traveled for female SD rats with 12.5 g-cm NYU contusive injuries at T10 in standard 10″ × 18″ cages. Digital video was made of 1 min for every 10 min throughout the dark (12 h) period. Video was made a 4 Hz using a Basler 602f camera and custom-built IR lighting. Distance was determined using MaxTraq by Innovision Systems for three nights from each 10 day bin. Data is shown as mean ± SD.

Prompted by our many observations that suggest in-cage retraining (for locomotion), we recently developed a system to monitor distance traveled using overhead video cameras running at 4 Hz and infrared lighting. We record activity for 1 min every 10 min and discovered that uninjured female Sprague Dawley rats walk more than 250 m each night when housed in pairs and that both cage size and single/double housing dramatically influences in-cage distance walked. These differences continue after injury as can be seen in Figure 4 showing the results for three animals that received 12.5 g-cm NYU contusion injuries at T10. Recordings were made for 3 days in each 10 day bin and animals were housed in standard 19” × 8” (48 cm × 20 cm) cages. Individually housed animals walk less despite having more room on a per animal basis. We have also found that cage size influences distance walked, but not to the same extent as having a cage mate. It should also be considered that the presence/absence of a cage-mate and/or an enriched environment might alter not only the distance walked and subsequent locomotor recovery (Lankhorst et al., 2001), but also might expose animals to different motor tasks like those associated with play or grooming another animal.

Lastly, activity can be influenced by pharmacological treatment, thus indirectly influencing functional outcome. A classical example is the administration of Rolipram, which is commonly used to increase intracellular cAMP levels. The fact that Rolipram also acutely decreases an animal’s activity level is frequently ignored (Wachtel, 1982; Silvestre et al., 1999).

### Variability between testing days

**Figure 5:**
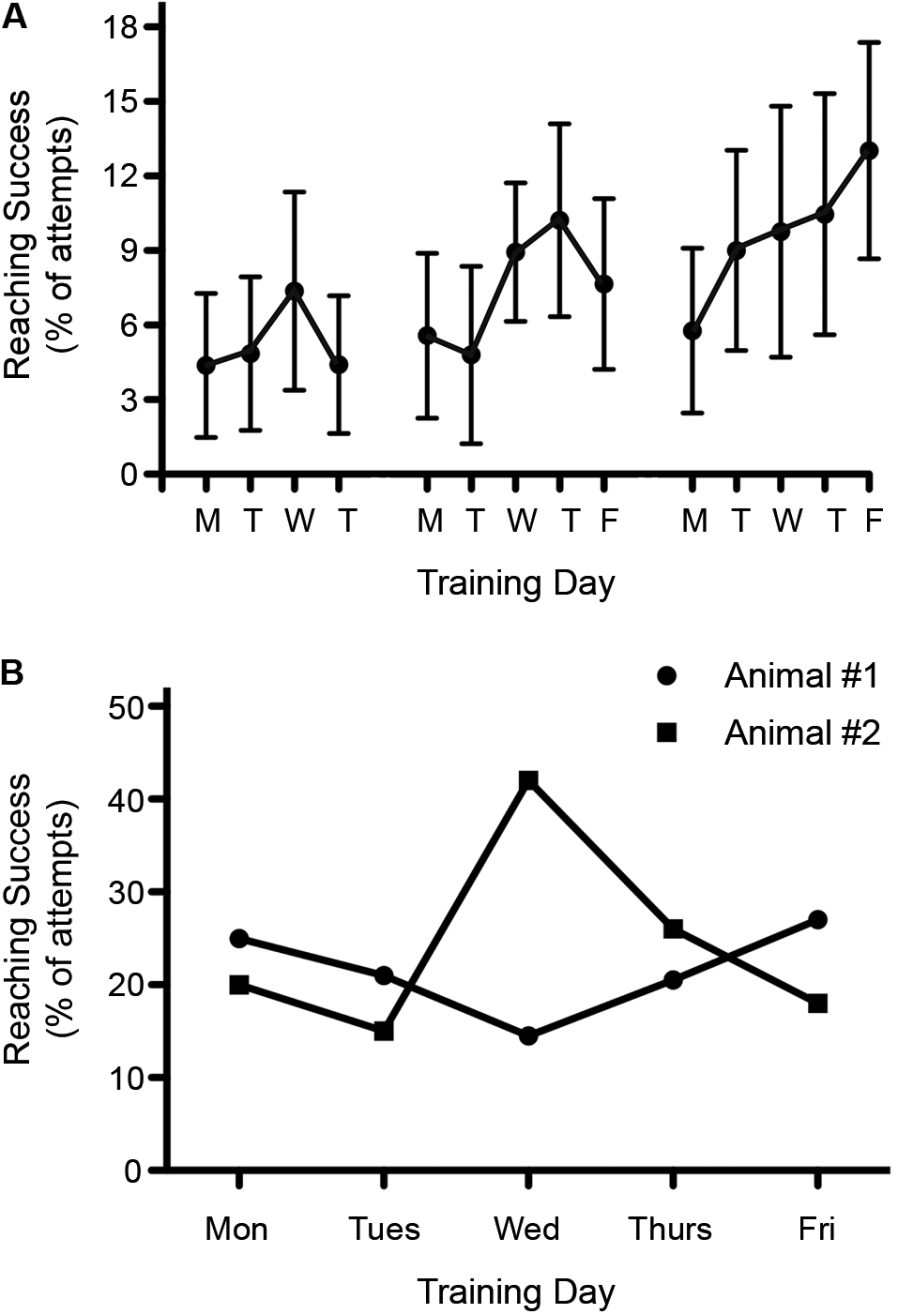
**Variability in reaching success throughout the week. (A)** Reaching recovery was assessed following a C4 dorsolateral quadrant spinal cord lesion. Success during rehabilitative training was found to be lowest on Mondays. Data is shown as mean ± SEM, *n* = 10. **(B)** Reaching success varied throughout the week among individual rats. Graph shows the pre-injury reaching performance for two different rats over the course of one week.

Significant variability in outcome measures is not only found between animals but also for individual animals tested on different days. This is especially significant for tests that are sensitive to factors such as stress, motivation, appetite etc. A good example is reaching for food items, whereas behaviors such as open field locomotion are less affected. A case in point: we have observed that performance in a skilled reaching task seems to vary depending on the day of the week. We have found that reaching attempts as well as reaching success are often lowest on Mondays, after rats are not trained for 2 days (Figure 5A). The reasons for this still remain unclear but may involve changes in the daily routine of the animals, how the weekend and weekday routines change in the animal facility, or in the variation in the motivation of the trainers throughout the week.

When testing single pellet reaching, the variability between testing days can easily exceed possible treatment effects (or result in false positive outcomes). Consistent with this possibility is the fact that we have observed extremely high variability over 5 days of single pellet training among individual rats (Figure 5B). To reduce variability, one could average the data over a few days or use the best result out of three or more measurements. Either of these approaches can be justified because an animal’s best performance still indicates their true ability, but there are plenty of reasons why performance might decline, including motivation, stress or time of testing.

### Floor and ceiling effects

Before behavioral tests are chosen to quantify recovery for a specific experiment, the functional consequences of the chosen lesion should be examined. If the behavioral tests are too challenging they might not allow sufficient success in the task, resulting in a floor effect. In this situation the control group data will not be normally distributed and the true ability of the animals will not be assessed. As a consequence, any treatment-induced recovery will be underestimated. The other extreme is a test that is too easy resulting in a ceiling effect, where resolution is not possible because the animals are not easily distinguished from pre-injury performance. This is particularly problematic if untreated animals get close to pre-injury performance levels allowing only a small margin for recovery. In such a scenario, treatment effects are unlikely to be revealed due to the commonly observed variability. However, a ceiling effect can also be found when non-linear scores or scales are used resulting in functional barriers (recovery plateaus at these points in the scale because the difference between two points on the scale are drastic). Sometimes, results are reported where a treated group initially improves more rapidly than the control group, however, over time the two groups plateau at a similar level. In such a case it remains unclear whether a functional, mid-scale ceiling effect limited the ability to detect recovery.

In conclusion, detecting a treatment effect following SCI in animal models is not as straightforward as it appears. There is a delicate interplay between lesion characteristics and the chosen behavioral tests that will allow the detection of a treatment effect.

## Rehabilitative training versus regular testing

Following SCI in the clinical setting, rehabilitative training is (and likely will remain) an inherent part of any treatment regimen. In animal models, evidence has accumulated to suggest that task specific training is also an essential part of rehabilitation in order to translate treatments designed to promote axonal regeneration or neuroplasticity into recovery. This triggers the question whether rehabilitative training should also be a fundamental component of functional testing in animal models, and at what point regular testing turns into rehabilitative training. For example, in a study by Raineteau et al. (2001), rats were tested 3–4 times a week, which resulted in one of the strongest treatment effects of the IN-1 antibody. Training could possibly also affect a pharmacological treatment (or the other way around) and the timing of training could be critical to the overall outcome (Maier et al., 2009). The opposite has also been reported where training was essential to unravel a drug effect on functional recovery (García-Alías et al., 2009; Weishaupt et al., 2013). Lastly, the current assumption is that training effects after SCI are task specific, which has implications for pharmacological treatments in that their effectiveness may be limited to certain trained tasks, rather than allowing a “broader” recovery. Thus, using a battery of tests might not necessarily reflect the benefits of a treatment.

## Conclusion

From a clinical aspect, functional recovery is the most important outcome measure of any SCI treatment. However, the evaluation of recovery in animal models is not as straightforward as is often believed, likely resulting in the frequent occurrence of false positive and negative treatment effects. Lesion models, outcome measures and the hypothesized effect size of potential treatments have to be carefully chosen, and the reduction of variability has to be a high priority.

So, how do we address all these challenges in behavioral testing in animal models of SCI? Here are a few suggestions:

1. Choose lesion models according to many factors including rat strain, the available tests, the potential treatment effect size, and mechanism of the treatment.
2. Perform pilot experiments to allow adjustments to be made (lesion and tests including training/testing intensity) for successful testing for each given question. Avoid ceiling and floor effects.
3. Reduce variability, use a single tester, remove animals to secluded environment, use best performance over more than one testing day.
4. Add regular testing/training in one task, but perform final testing in a larger set of tasks evaluating different aspects of recovery and possible side effects.
5. Involve qualitative and quantitative approaches to understand the mechanisms of the observed recovery.
6. Observe your animals for changes that are not captured in your test(s). Obtain an all-encompassing picture of the recovery beyond motor function. The pattern of recovery may well be as important as the amount of recovery.

## Conflict of interest statement

The authors declare that the research was conducted in the absence of any commercial or financial relationships that could be construed as a potential conflict of interest.
